# Knowledge, Attitudes and Practices Regarding Diabetes in the General Population: A Cross-Sectional Study from Pakistan

**DOI:** 10.3390/ijerph15091906

**Published:** 2018-09-02

**Authors:** Ali Hassan Gillani, Fakir Mohammad Amirul Islam, Khezar Hayat, Naveel Atif, Caijun Yang, Jie Chang, Zhan Qu, Yu Fang

**Affiliations:** 1 Department of Pharmacy Administration and Clinical Pharmacy, School of pharmacy Xi’an Jiaotong University, Xi’an 710061, China; hassangillaniali@yahoo.com (A.H.G.); khezar.hayat@uvas.edu.pk (K.H.); naveelatif69@gmail.com (N.A.); yangcj@mail.xjtu.edu.cn (C.Y.); jiechang@mail.xjtu.edu.cn (J.C.); 2 Center for Drug Safety and Policy Research, Xian Jiaotong University, Xi’an 710061, China; 3 Shaanxi Centre for Health Reform and Development Research, Xi’an 710061, China; 4 Department of Statistics, Data Science and Epidemiology, Faculty of Health, Arts and Design, Swinburne University of Technology, Melbourne VIC 3122, Australia; fislam@swin.edu.au; 5 Organization for Rural Community Development, Dariapur, Narail 7500, Bangladesh; 6 Institute of Pharmaceutical Sciences, University of Veterinary and Animal Sciences, Lahore 54000, Pakistan; 7 School of Nursing, Health Science center, College of Medicine, Xian Jiaotong University, Xi’an 710061, China; zhanqu.xjtu@gmail.com

**Keywords:** knowledge, attitude, practice, diabetes mellitus, general population, Pakistan

## Abstract

*Background*: Low knowledge about diabetes risk factors coupled with high disease prevalence is common in low-resource countries. This study evaluated diabetes-related knowledge, attitudes, and practices in the general population in Punjab (Pakistan). *Methods*: A cross-sectional study was conducted in five districts in Punjab from January to March 2017. Data were collected from 2019 adults aged 18–90 years through face-to-face interviews using a semi-structured questionnaire. The total knowledge score ranged from 0–9; a score ≥6 was considered adequate diabetes awareness. Descriptive statistics, chi-square tests, and linear and binary logistic regression were used for the analyses. *Results*: Respondents’ mean age was 32.92 ± 11.4 years. In total, 85.9% of respondents had heard of diabetes, and 30.1% knew about the glucose tolerance test. We found 2.3% of respondents scored zero for diabetes knowledge, 11.3% scored 9, and 47.4% scored ≥6 (adequate awareness). Being female (β = 0.37, 95% confidence interval [CI]: 0.16, 0.05; *p* = 0.001), socioeconomic status (β = 0.24, 95% CI: 0.12, 0.36; *p* < 0.001), being diabetic (β = 0.82, 95% CI: 0.53, 1.10; *p* < 0.001), and higher education (β = 0.25, 95% CI: 0.17, 0.33; *p* < 0.001) were significantly associated with knowledge score. Respondents with high socioeconomic status showed significantly higher positive attitudes compared with those with low socioeconomic status (adjusted odds ratio 1.57, 95% CI: 1.12, 2.24). Only 8.7% (30/343) of those diagnosed with diabetes had never undergone blood glucose screening since diagnosis. *Conclusions*: Knowledge of diabetes risk factors, management, and care is low in Pakistan’s general population. Targeted public education programs should be instigated at a national level to increase understanding of diabetes prevention and treatment.

## 1. Introduction

More than two-thirds (70%) of patients with diabetes mellitus (DM) reside in lower middle-income countries [1]. The World Bank classifies Pakistan as a lower middle-income country [2]. Pakistanis currently ranked 6th in terms of DM cases globally [3], with a DM prevalence of 6.9% [4]. In 2020, Pakistan is projected to become the 4th leading country in the number of patients with DM [3]. This increase may be attributed to altered lifestyles and the subtle nature of the disease. A large proportion of Pakistan’s population remain undiagnosed until manifestation of co-morbidities (i.e., eye disease, renal disorder [5]), with a reported 7.9 million people having impaired glucose tolerance [1].

Evidence suggests that various socio-demographic determinants, including poor health knowledge, account for the epidemiologic shift of DM in lower middle-income countries [6,7]. Growing evidence from knowledge, attitude, and practice (KAP) studies indicates there is an urgent need to enhance DM awareness, early diagnosis, control of risk factors, and disease management [8,9]. Most KAP studies have been conducted among diagnosed or newly diagnosed patients with DM attending hospitals or healthcare centers [6,7,9]. However, KAP regarding DM and related complications is seldom investigated in the general population. Therefore, there is a paucity of research evidence, particularly from remote areas.

Over the last decade, some studies were conducted to evaluate the occurrence and risk factors of DM in rural and urban Pakistan [10,11] and others have evaluated co-morbidities associated with diabetes [12,13]. However, no study has estimated the KAP regarding DM, along with its treatment and management and risk factors in the general population of Punjab. An exception is a study that evaluated DM awareness among 383 respondents from the general population of Bahawalpur and found that the majority (80.4%) were enthusiastic about attending a DM awareness program [14]. Evidence from Mongolia, another low-resource country, reported that in 2010, 20% of the general population had never heard about DM [6]. 

An Australian randomized controlled trial suggested that incorrect knowledge about DM risk factors and motivation to make lifestyle changes were significantly associated with diet modifications and exercise habits. That study also reported a strong association between lifestyle modifications and reduction in waist circumference, body mass index, and blood glucose level (BGL) [15,16]. However, these factors still remain challenges for developing countries, including Pakistan. In 2004, Pakistan formed a national action plan to prevent non-communicable diseases (NCD). The main agenda in terms of DM was construction of a population-based NCD surveillance system and integrated public health program. This aimed to integrate DM prevention and ensure availability of anti-diabetics at all levels of healthcare [17]. Despite the high growth in NCD, the response by the National Action Plan Committee was slow, and a World Health Organization (WHO) progress report found Pakistan lagged behind the national NCD target level with a 25% increase in NCD-related deaths [18]. In this context, no research has been conducted to evaluate the DM-related knowledge and attitudes in the general population. Such research is important to clarify the knowledge levels in the general population and evaluate the effect of increased knowledge on the prevalence of DM. Therefore, our study aimed to investigate KAP regarding DM and clarify factors associated with KAP among the general population in five districts in Punjab, Pakistan.

## 2. Materials and Methods

### 2.1. Study Areas

**Figure 1:**
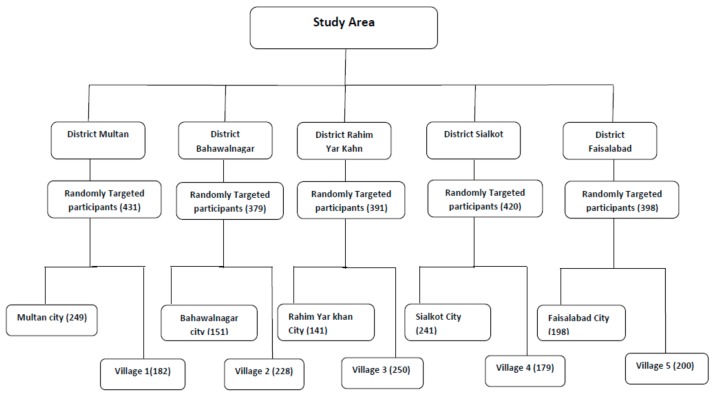
The number of individuals selected from each district.

Pakistan has five provinces that are further divided into divisions, districts, and tehsils. Villages comprise the lowest socio-demographic unit. Punjab occupies 26% of the total landmass of Pakistan, and comprises nine divisions and 36 districts; 60% of Pakistan’s population resides in Punjab [19,20]. Five districts (Rahim Yar khan, Multan, Bahawalnagar, Faisalabad, and Sialkot) were selected for this study. From each of these districts, we selected the district headquarter city and one village. The village selection from each district was random. Details of the number of individuals recruited from each city and village are presented in Figure 1. 

### 2.2. Study Population and Recruitment

Inclusion criteria for respondents from selected cities and villages were individuals aged over of 18 years (regardless of religion and geographical locality), without mental illness, and willing to participate in this study. Cities were divided into four geographical parts (east, west, south, and north), and an almost equal number of households were randomly targeted from each geographical location. Face-to-face interviews were conducted with willing respondents by the data collector following door-to-door visits. We interviewed one resident from each household that fulfilled the inclusion criteria. Selection of respondents from among multiple household residents was made by a lottery method. The same data collection procedure was adopted for selected villages, but they were not divided into different geographical regions. Exclusion criteria were those aged <18 or >90 years, with any mental illness, or who were not willing to participate.

### 2.3. KAP Questionnaire and Validation

A questionnaire was developed by the present researchers to collect data. The questions covered demographic information, lifestyle factors, and DM knowledge, risk factors, and disease management (including medication behavior). Questions included in the knowledge section were drawn from the Chennai Urban Rural Epidemiology Study [21] and the Aus Diab Health Knowledge, Attitudes, and Practices Questionnaire [22]. Knowledge items from Mohan et al. [21] were also used, because Indian and Pakistani people have similar lifestyles. The question regarding smoking cessation and DM control was taken from the Aus Diab study [22]. Respondents’ DM status was assessed by asking if he/she had ever been diagnosed with DM or was currently taking medication for DM. Answers to the knowledge questions were dichotomized (Yes/No). Table 1 presents the content and response options for the questionnaire used in this study. The questionnaire included nine knowledge items, with a score of “1” awarded for correct answers and “0” for incorrect answers. The total knowledge score ranged from 0–9; a score of ≥6 was considered to reflect adequate awareness.

Responses to the item evaluating attitudes toward DM treatment were also categorical (Yes, No, Don’t know). Respondents were asked: “If you, your family, or friends had diabetes would they seek treatment?” Responses of “yes” were considered to reflect a positive attitude. The practice item was specifically for those who had been diagnosed with DM, and was assessed by asking: (1) “Are you currently taking the diabetes medication?” and (2) “How often do you go for BGL screening?” The response to the first question was dichotomous (Yes/No), and that for the second question was categorized as: 1 = more than once a year (frequently), 2 = at least once in a year (regularly), 3 = whenever I think my diabetes is worse and 4 = never since diagnosed.

Socio-demographic data included age (categorized as 18–30 years, 31–45 years, 46–60 years, and ≥60 years) gender, education status (categorized as nil, primary school 1–5 year schooling, high school 6–10 Year, intermediate 10–12 year, and above intermediate), residence (urban or rural) and socioeconomic status (SES (Socioeconomic Status): categorized as insufficient funds for the whole year = 1, insufficient funds sometimes = 2, balance = 3, and sufficient funds most times = 4). To check respondents’ hypertension status, a research assistant took two consecutive blood pressure readings using a mercury or digital sphygmomanometer. We used the WHO definition for hypertension: “patients with systolic blood pressure 160 mmHg or more and/or diastolic blood pressure 95 mmHg or more, or if under ongoing treatment with antihypertensive drugs.” 

The questionnaire used in this study was pre-validated and used in the general rural population in Bangladesh [5]. The questionnaire was further checked for validity and consistency in our study population. Before starting this study, a pilot test was conducted with 20 individuals from each district in the selected areas. Minor modifications (e.g., rephrasing) were made based on this pilot study.

### 2.4. Ethics Approval and Consent to Participate

This study was performed according to tenets of Helsinki Declaration and also approved by ethics committee for Medical Research of Xi’an Jiaotong University, Shaanxi, China (Reference; DIAB-98-2016). Furthermore, this study is also approved by the ethics committee of Islamia University of Bahawalpur, Punjab, Pakistan. Written approval was obtained from those who were able to read and sign the consent form. Investigator signed the consent form after seeking permission from uneducated persons who were unable to understand the consent form. Participants were made completely aware about the liberty to leave the study at any stage. 

### 2.5. Training of Data Collectors and Quality Assurance of Data

Five teams, each containing two research assistants, were given extensive training by the principal investigator (PI). Training involved the following aspects: (1) Presenting a brief introduction of the study purpose to respondent; (2) Conducting face-to-face interviews; (3) Coping with difficulties during the data collection. Training was carried out for 3 days, with a demonstration given by the PI. Trainees then conducted pilot study in each of their respective districts and were observed for their interviewing skills by PI. After completion of training each team was allotted one district and send out to collect the data.

PI in order to maintain the data quality and validity carried out spot investigations; regular checks review and ensure the completeness of the filled questionnaires. In the meantime the PI synchronized the overall study.

### 2.6. Statistical Analysis

Descriptive statistics were used for all demographic variables (gender, age, locality, SES, occupation, and education). Associations between knowledge scores and demographic characteristics were evaluated using regression analysis. Beta coefficients with 95% confidence intervals (CI) were calculated after adjusting for gender, age, residence, SES, education, being hypertensive, and being diabetic. All demographic characteristics and other factors underwent multivariate regression analyses for knowledge. For attitude toward DM treatment (binary outcome), associations between the variables were investigated using binary logistic regression (adjusted odds ratio (AOR), 95%CI). Chi-square tests were used to describe the statistical differences between the practice items and demographic information for those diagnosed with DM. Responses related to DM practice were investigated using descriptive statistics. All statistical analyses were conducted using SPSS (SPSS Inc, version 18, SPSS Inc., version 18, IBM, Chicago, IL, USA). 

## 3. Results

### 3.1. Demographic Data

Of 2073 potential respondents approached, 2019 completed an interview, giving a response rate of 97.4%; 94.8% were women (710/749) and 98.8% were men (1309/1324). The mean age was 32.92 ± 11.4 years (men 35.0 ± 12.2 years, women 28.9 ± 8.4 years), and the age range was 18–86 years. Among the 2019 respondents, 64.8% were men, 36.7% (*n* = 740) had an education level above intermediate (45.1% women and 54.9% men), and 11% (*n* = 222) had insufficient funds most of the time in the past year. A majority of respondents were students (41.0%) and 13.7% were farmers. In the group aged ≥60 years, women were reluctant to participate in this study (4.2%), meaning men comprised 95.8% of this age group. All respondents completed an interview and responded to all questions (Table 2).

### 3.2. Knowledge Score and Association with Demographic Characteristics

There was variation in responses for knowledge items. Only 30% of respondents knew what a glucose tolerance test was, although 87% had heard of DM. The urban population was more aware of how to measure DM than the rural population (48.5% vs. 45.7%), and more urban residents knew that DM is a genetic disease compared with rural residents (61.5% vs. 49.1%). Respondents in the younger age groups had comparatively higher knowledge on five knowledge items than those aged ≥60 years. For example, a significantly higher proportion (55.9%) of respondents aged 31–44 years knew that reducing carbohydrate intake can control diabetes compared with those aged ≥60 years (44.6%). In addition, scores for six knowledge items were significantly higher among respondents who had sufficient funds for most of the past year than those who had insufficient funds for the whole year (*p* ≤ 0.05) (Table 3 and Table 4). Only 2.3% (46/2019) of respondents were not aware of any knowledge items, and 11.3% (228/2019) were aware of all knowledge items. An adequate awareness (score ≥ 6) was observed among 47.4% (*n* = 957) of respondents.

### 3.3. Multivariate Analysis for Knowledge Scores

After adjusting for all variables in the multivariable model, women were significantly more aware of DM than males. Similarly, SES (β = 0.24, 95% CI: 0.12, 0.36; *p* < 0.001) and having diabetes (β = 0.82, 95% CI: 0.53, 1.10; *p* < 0.001) were significantly associated with knowledge score in the multivariable regression analysis (Table 5).

### 3.4. Attitudes toward DM Treatment

After adjusting for all demographic variables in the multivariable model, respondents with sufficient funds for most of the past year showed a significantly higher positive attitude towards DM treatment (AOR 1.57, 95% CI: 1.11, 2.23; *p* = 0.01) than other SES groups (Table 6). Gender, residence (urban/rural), and education level were not significantly associated with attitudes toward DM treatment.

### 3.5. Practice

There were 343 (17%) respondents that had been diagnosed with diabetes. Of these, 24.8% (*n* = 85) checked their BGL more than once a year, 36.4% (*n* = 125) checked at least once a year, and 8.7% (*n* = 30) had never undergone BGL screening since their DM diagnosis. There were significant differences among all demographic variables (*p* ≤ 0.05) for DM practice, except residence (*p* > 0.05). For example, 42.4% of the rural population checked their BGL more than once a year, 47.2% screened at least once a year, and 70% had never screened after diagnosis (Table 7). 

## 4. Discussion

Previous studies reported that DM management and care are strongly related to adequate knowledge, and there is a correlation between DM knowledge and hemoglobin A1c level [23,24,25]. The present study demonstrated unsatisfactory outcomes in terms of DM knowledge, with only 11.3% of respondents knowing all knowledge items and 47.4% having adequate knowledge. Our study also suggested there were positive practices regarding BGL among those diagnosed with DM, as only a minority (8.7%) of diagnosed patients had never had their BGL checked since diagnosis.

A previous study in Bangladesh (2012) found that 82% of people had a basic level of DM awareness [26], which was considerably higher than in our study. This disparity may be explained by the previous study using the Diabetes Knowledge Test, a tool validated by the University of Michigan, whereas our tool was researcher-constructed. Similarly, another study from Bangladesh found that 62% of the population had an adequate knowledge level [5], which was also higher than our result.

Our study showed that knowledge regarding DM measuring parameters in Punjab was low, as only 30.2% of respondents know about the glucose tolerance test and 47.1% knew about how to measure diabetes. A study conducted in Croatia among pregnant women found that 42% of participants demonstrated a high level of knowledge about the glucose tolerance test, which was consistent with our study, although the study populations differed [27]. Our results were considerably higher than those from Bangladesh, where only 4% of the population knew what glucose tolerance test was and 9% knew how to measure DM [5]. We found that knowledge of DM control and management was appropriate (i.e., 70.2% agreed DM can be managed by exercise, 78.4% thought controlling sweets would control DM, and 60.6% believed avoiding smoking will help control DM). Previous studies indicated that poor DM knowledge was evident in developed and developing countries [9,14,28]. A study conducted in Gujrat, India, among patients with DM reported that 51% of patients believed exercise helped manage DM and 75% reported diet management as an integral part of disease management, but only 7% associated quitting smoking with DM management [28]. Similarly, a study among 575 patients with DM in the United Arab Emirates (UAE) found a majority (60%) of patients thought DM can be caused by excessive sugar intake [9], which was lower than the 78.4% in our study who agreed controlling sugar will control diabetes. Another study in Pakistan demonstrated that only 23.5% of the general population agreed DM can affect other organs, and 35.5% thought people with DM should go for regular eye checks [12]. In our study, 49.5% of respondents agreed that diabetes can cause eye disease, which was significantly higher than the previous study and consistent with the study by Memon et al. that reported 56.9% of the general population knew DM affected eyes [29]. Half of our study population (51.6%) stated that genetic factors may increase DM risk, which was lower than a Sri Lankan study in which 73% of respondents reported a family history of DM as a risk factor [30].

In relation to variables associated with KAP, a study by Al-Maskari et al. among patients with DM reported that age, gender, and SES were related to DM knowledge, and observed a higher knowledge score among males than females (*p* < 0.001) [9]. That study also found there was a significant difference between knowledge scores of postgraduate (19.67) and undergraduate (14.74) respondents (*p* < 0.001) [9]. Our results were consistent with that study. Similarly, a study by Islam et al. showed significant associations for all demographic variables (including DM status) with knowledge scores [5]; similar outcomes were observed in our study, except a non-significant association for age. Significantly higher knowledge scores among males than females may be related to a higher level of education among males. Among the 740 respondents with an education above intermediate in our study, 406 (54.9%) were men and 334 (45.1%) were women (data not shown). 

A positive attitude toward seeking healthcare for DM was strongly associated with having sufficient income for most of time compared with an insufficient income for the whole year (AOR 1.570, 95% CI: 1.108, 2.227; *p* = 0.011), with similar results reported in the study in Bangladesh [5]. The similarity in these results may be attributable to the similar SES and income status between the two studies. In contrast, the study among patients with DM in the UAE did not report associations between attitudes and income/SES, which may be explained by the large difference between the gross domestic product (GDP) of Pakistan and the UAE. In 2015, the GDP per capita for Pakistan was 1142.7 USD [31] whereas that for the UAE was 39,313.3 USD [32]; therefore, an assumption can be made that attitude toward seeking treatment is related to the income of the population.

BGL monitoring after diagnosis among people with DM was unexpectedly high, and only 8.7% of population had never been screened since their diagnosis. This result contradicts that of the study by Islam et al. that showed more than 50% of respondents had never performed screening since diagnosis [5]. Our results were somewhat similar to those of a study in Karachi that showed 87.5% of diabetic and 9.3% of non-diabetic participants had regular DM checking habits [29]. Our results were inconsistent with a study by Kassahun et al. involving 594 patients with DM in Bale zone, Ethiopia, that reported 40.6% of respondents never checked their BGL and 7.1% were engaged in frequent BGL observation [33]. The difference in results may be attributed to Ethiopia’s low SES.

On World Diabetes Day, the Government of Pakistan organizes walks, education programs, and free camps; however, no such actions are taken to educate those living in rural areas. It is crucial to enhance basic DM knowledge among the general population, especially in rural areas. National-level education and health intervention programs should be instigated and augmented. Properly designed and planed interventions among patients with DM should be conducted at the physician- and community pharmacy-level to ensure patients are aware and vigilant regarding disease management. Educational campaigns should focus on dietary and lifestyle modifications, and regular BGL screening in addition to risk factors. Diabetes educators should be part of the healthcare team, and should be supported to organize health education programs in both urban and rural areas. BGL screening programs should be initiated at basic health units to diagnose at the pre-diabetes level and support timely control of the disease. The government should strictly follow the WHO recommendations on NCD management, and implement and reinforce the national action plan agenda for NCD control.

## 5. Limitations

The first drawback of this study was the selection of the district was random and similarly village in selected districts were targeted randomly. However, efforts were made to collect the data from different geographic region of Punjab. Secondly, the sample mainly consist of the male students which may indicate the biasness in the random selection of the respondents. This could be answered that we do not just randomly pick up the participant from multiple members of one house, we adopted a well developed and validated lottery method which gives the same probability to each willing individual to be recruited in the study. Students or the educated community understands the research activities and show more willingness to such surveys. Thirdly, the sample size may not be representative of the whole province however the respondents were selected randomly and from wide geographical area so can be considered as representative sample. Fourthly, our sample consist of 2/3 males whereas the census show that percentage of females more than males this creates unequal distribution of sample and sample may not be true representative. This effect is due to reluctant behavior of female in the participation also the education level of the females is less which is also hindering factor to be part of an epidemiological study. This could be overcome by using female research assistants to interview females. Fifth, we selected only one individual from a house even if multiple members were willing. This effect reduces the repetition of the same responses as observed in the previous studies that when we approach multiple members the answers are almost the same or even sometimes they ask us to copy the answers from other members questionnaire. Sixth, this study showed much higher prevalence (17%) of the patients with diabetes as compared to national level (7.1%). Again this is not intentional and respondents were selected randomly. Lastly this study focused in Punjab only so cannot be generalized to the whole country. Nevertheless, as the first survey of its kind from the most populated region in Pakistan, the current study provided us a clearer picture of the KAP of DM among general public in Pakistan.

## 6. Conclusions

In conclusion, knowledge regarding risk factors, management, and care of DM in the general population of Punjab (Pakistan) is low. There is an increase in the level of knowledge with age, and the urban community show more knowledge than rural residents. Intervention programs should focus on younger aged and rural populations to offer the most benefit to the community. People that have sufficient financial resources tend to show better attitudes toward seeking treatment. Those with fewer resources should be provided with free screening services and encouraged to attend. In addition, women should be encouraged to have regular BGL checks to improve good management practices.

## Figures and Tables

**Table 1:** Awareness of diabetes and its risk factors of 2019 adults interviewed in the cross-sectional study.

Knowledge Related Item	Response, Yes	
	*N*	(%)
Have you ever heard of diabetes?	1759	87.1
Do you know what glucose tolerance test is?	609	30.2
Do you know how to measure diabetes?	950	47.0
Do you know diabetes can cause eye disease?	1007	49.9
Do you know diabetes is a genetic disease?	1113	55.1
Do you know exercise can be helpful to prevent diabetes?	1416	70.1
Do you know that reducing carbohydrate intake can reduce diabetes?	1250	61.9
Do you know that reducing sugar intake, reduce diabetes?	1583	78.4
Do you know diabetes can be controlled by avoiding smoking?	1224	60.6

**Table 2:** Demographic details.

Variables		Number (*n*)	Percentage (%)
Age (32.92 ± 11.4)	18–30	1134	56.2
	31–45	596	29.5
	46–60	242	12.0
	More than 60	47	2.3
Gender	Male	1309	64.8
	Female	710	35.2
Residence	Urban	980	48.5
	Rural	1039	51.5
Education	Nil	62	3.1
	1–5	420	20.8
	6–10	464	23.0
	10–12	333	16.5
	More than Intermediate	740	36.6
Occupation	Student	828	41.0
	Farmer (kisan)	276	13.8
	Laborer	184	9.1
	Housewife	218	10.8
	Teacher	194	9.6
	Business man	222	11.0
	Other	53	2.6
	None	43	2.1
Socioeconomic Status (SES)	Insufficient funds for whole year	222	11.0
	Insufficient for some time	462	22.9
	Balance	1122	55.6
	Sufficient funds for most of times	213	10.5

**Table 3:** General Knowledge (known or heard about) of Diabetes and Common Eye Disease by Gender, Religion, Age, Education.

Questions	Gender		*p*	Age Group		*p*	Education Status					*p*
	Male *n* = 1309 (%)	Female *n* = 710 (%)		18–30 *n* = 1134 (%)	>60 *n* = 47 (%)		Nil *n* = 62 (%)	1–5 *n* = 420 (%)	6–10 *n* = 464 (%)	10–12 *n* = 333 (%)	Greater than Inter *n* = 740 (%)	
Q1: Heard of diabetes?	1146 (87)	613 (86)	0.431	984 (87)	45 (96)	<0.001	57 (92)	346 (82)	382 (82)	301 (90)	673 (91)	<0.001
Q2: What glucose tolerance test is?	341 (26)	268 (38)	<0.001	386 (34)	14 (30)	<0.001	10 (16)	85 (20)	83 (18)	128 (38)	203 (27)	<0.001
Q3: How to measure diabetes?	579 (44)	339 (48)	0.001	572 (50)	20 (42)	0.02	17 (27)	166 (39)	153 (33)	169 (51)	445 (60)	<0.001
Q4: Diabetes can cause eye disease?	616 (47)	391 (55)	0.001	549 (48)	21 (45)	0.06	21 (34)	198 (47)	202 (43)	192 (58)	394 (53)	<0.001
Q5: Diabetes is a genetic disease?	676 (52)	431 (61)	<0.001	645 (57)	22 (47)	0.235	26 (42)	222 (53)	225 (48)	214 (64)	426 (58)	<0.001
Q6: Exercise can be helpful to prevent diabetes?	893 (68)	523 (74)	0.009	801 (71)	30 (64)	0.776	29 (47)	271 (64)	299 (64)	246 (74)	571 (77)	<0.001
Q7: Reducing carbohydrate intake can reduce diabetes?	763 (58)	487 (68)	<0.001	763 (67)	21 (45)	<0.001	24 (39)	208 (49)	247 (53)	205 (62)	566 (76)	<0.001
Q8: Reducing sugar intake, reduce diabetes?	1043 (80)	540 (76)	0.067	890 (78)	37 (79)	0.03	50 (81)	305 (73)	362 (78)	260 (78)	606 (82)	0.008
Q9: can be controlled by avoiding smoking?	759 (58)	465 (65)	0.001	676 (60)	31 (66)	0.417	28 (45)	242 (58)	244 (53)	207 (62)	503 (68.0)	<0.001

**Table 4:** General Knowledge (known or heard about) of Diabetes and Common Eye Disease by Socio-economic Condition.

Questions	Locality		*p*	SES (Socioeconomic Status)				*p*
	Urban *n* = 980 (%)	Rural *n* = 1039 (%)		Insufficient Funds for Whole Year *n* = 222 (%)	Insufficient for Some Time *n* = 462 (%)	Balance *n* = 1122 (%)	Sufficient Funds for Most of Times *n* = 213 (%)	
Q1: Heard of diabetes?	830 (85)	929 (89)	0.002	179 (81)	358 (77)	1029 (92)	193 (91)	<0.001
Q2: What glucose tolerance test is?	330 (34)	279 (27)	0.001	78 (35)	122 (26)	350 (31)	59 (28)	0.07
Q3: How to measure diabetes?	475 (48)	475 (46)	0.223	108 (49)	205 (44)	550 (49)	87 (41)	0.083
Q4: Diabetes can cause eye disease?	502 (51)	505 (49)	0.23	111 (50)	199 (43)	577 (51)	120 (56)	0.004
Q5: Diabetes is a genetic disease?	603 (61)	510 (49)	<0.00	116 (52)	279 (60)	620 (55)	107 (50)	0.159
Q6: Exercise can be helpful to prevent diabetes	714 (73)	702 (68)	0.008	144 (65)	307 (66)	818 (73)	147 (69)	0.16
Q7: Reducing carbohydrate intake can reduce diabetes?	637 (65)	613 (59)	0.005	134 (60)	237 (51)	744 (66)	135 (63)	<0.001
Q8: Reducing sugar intake, reduce diabetes?	765 (78)	818 (79)	0.748	151 (68)	347 (75)	910 (81)	175 (82)	<0.00
Q9: can be controlled by avoiding Smoking?	567 (58)	657 (63)	0.015	120 (54)	250 (54)	722 (64)	131 (61)	<0.001

**Table 5:** Demographic association with knowledge score.

Variables	β (95% CI) *	*p*	β (95% CI) **	*p*
Age (Reference 18 year)	−0.015 (−0.024, −0.006)	0.001	−0.005 (−0.015, 0.004)	0.264
Females vs. Male	0.555 (0.348, 0.763)	<0.001	0.374 (0.162, 0.586)	0.001
Urban vs. Rural	−0.254 (−0.453, −0.054)	0.013	−0.168 (−0.366, 0.030)	0.097
Education (Ref no schooling)	0.253 (0.384, 0.593)	<0.001	0.255 (0.176, 0.335)	<0.001
SES (Ref Insufficient funds for whole year)	0.240 (0.117, 0.363)	<0.001	0.243 (0.120, 0.366)	<0.001
Patients with Diabetes	0.759 (0.495, 1.022)	<0.001	0.823 (0.538, 1.109)	<0.001
Hypertensive patients	0.384 (0.162, 0.606)	<0.001	0.220 (−0.017, 0.457)	0.069

* Unadjusted β (95%CI); ** multivariable adjusted β (95%CI) adjusted for age, gender, residence level of education, SES, being diabetic and being hypertensive.

**Table 6:** Association of attitude (should they seek treatment?) with demographic variables.

Variables		Don’t Know/No	Yes	AOR (95%CI)	*p*
	Total	416 (%)	1603 (%)		
Age years	18–30	232 (20.4)	902 (79.6)	1	
	31–45	133 (22.3)	463 (77.7)	1.38 (0.68, 2.78)	0.37
	46–60	39 (16.1)	203 (83.9)	1.25 (0.62, 2.50)	0.53
	More than 60	12 (25.5)	35 (74.5)	1.78 (0.85, 3.77)	0.13
Gender	Male	267 (20.4)	1042 (79.6)	1	
	Female	149 (21.0)	561(79.0)	0.93 (0.73, 1.18)	0.54
Residence	Urban	195 (19.9)	785 (80.1)	1	
	Rural	221 (21.2)	818 (78.8)	0.89 (0.71,1.11)	0.31
Education	Nil	10 (16.1)	52 (83.9)	0.81 (0.39, 1.69)	0.58
	1–5	77 (18.3)	343 (81.7)	0.89 (0.43, 1.85)	0.76
	6–10	116 (25.0)	348 (75.0)	0.57 (0.28, 1.19)	0.14
	10–12	71 (21.3)	262 (78.7)	0.76 (0.36, 1.61)	0.47
	More than Intermediate	142 (19.2)	598 (80.8)	1	
Socioeconomic Status (SES)	Insufficient funds for whole year	63 (28.4)	159 (71.6)	1	
	Insufficient for some time	94 (20.3)	368 (79.7)	0.85 (0.55, 1.30)	0.45
	Balance	204 (18.2)	918 (81.8)	1.38 (0.93, 2.04)	0.11
	Sufficient funds for most of times	55 (25.8)	158 (74.2)	1.58 (1.12, 2.24)	0.01

**Table 7:** Association of demographics with frequency of blood glucose check since diagnosis in patients with diabetes (*n* = 343).

Variables		More than Once a Year *n* = 85 (%)	At Least Once in a Year *n* = 125 (%)	When You Think Your Diabetes Is *n* = 103 (%)	Never Since Diagnosed *n* = 30 (%)	*p*
Age Years	18–30	44 (51.8)	57 (45.6)	30 (29.1)	14 (46.7)	<0.001
	31–45	25 (29.4)	54 (43.2)	31 (30.1)	3 (10.0)	
	46–60	10 (11.8)	12 (9.6)	38 (36.9)	11 (36.7)	
	More than 60	6 (7.0)	2 (1.6)	4 (3.9)	2 (1.6)	
Gender	Male	62 (72.9)	66 (52.8)	73 (70.9)	27 (90.0)	<0.001
	Female	23 (27.1)	59 (47.2)	30 (29.1)	3 (10.0)	
Residence	Urban	49 (57.6)	66 (52.8)	47 (45.6)	9 (30.0)	0.057
	Rural	36 (42.4)	59 (47.2)	56 (54.4)	21 (70.0)	
Education	Nil	4 (4.7)	0 (0.0)	2 (1.9)	3 (10.0)	<0.001
	1–5	27 (31.8)	40 (32.0)	21 (20.5)	4 (13.3)	
	6–10	12 (14.1)	26 (20.8)	25 (24.3)	5 (16.7)	
	10–12	22 (25.9)	24 (19.2)	12 (11.6)	8 (26.7)	
	More than intermediate	20 (23.5)	35 (28.0)	43 (41.7)	10 (33.3)	
Socioeconomic Status (SES)	Insufficient funds for whole year	32 (37.6)	8 (6.4)	8 (7.8)	4 (13.3)	<0.001
	Insufficient for some time	27 (31.8)	68 (54.4)	26 (25.2)	9 (30.0)	
	Balance	22 (25.9)	49 (39.2)	65 (63.2)	15 (50.0)	
	Sufficient funds for most of times	4 (4.7)	0 (0.0)	4 (38.8)	2 (6.7)	
